# Fabrication and Characterization of Oxygen-Generating Polylactic Acid/Calcium Peroxide Composite Filaments for Bone Scaffolds

**DOI:** 10.3390/ph16040627

**Published:** 2023-04-20

**Authors:** Abdullah Mohammed, Abdu Saeed, Amr Elshaer, Ammar A. Melaibari, Adnan Memić, Hany Hassanin, Khamis Essa

**Affiliations:** 1 School of Engineering, University of Birmingham, Birmingham B15 2TT, UK; 2 Center of Nanotechnology, King Abdulaziz University, Jeddah 21589, Saudi Arabia; 3 Drug Discovery, Delivery and Patient Care, School of Life Sciences, Kingston University London, Kingston Upon Thames KT1 2EE, UK; 4 Department of Mechanical Engineering, King Abdulaziz University, Jeddah 21589, Saudi Arabia; 5 School of Engineering, Canterbury Christ Church University, Canterbury CT1 1QU, UK

**Keywords:** 3D printing filament, bone scaffold, tissue engineering, oxygen releases, poly lactic acid, calcium peroxide

## Abstract

The latest advancements in bone scaffold technology have introduced novel biomaterials that have the ability to generate oxygen when implanted, improving cell viability and tissue maturation. In this paper, we present a new oxygen-generating polylactic acid (PLA)/calcium peroxide (CPO) composite filament that can be used in 3D printing scaffolds. The composite material was prepared using a wet solution mixing method, followed by drying and hot melting extrusion. The concentration of calcium peroxide in the composite varied from 0% to 9%. The prepared filaments were characterized in terms of the presence of calcium peroxide, the generated oxygen release, porosity, and antibacterial activities. Data obtained from scanning electron microscopy and X-ray diffraction showed that the calcium peroxide remained stable in the composite. The maximum calcium and oxygen release was observed in filaments with a 6% calcium peroxide content. In addition, bacterial inhibition was achieved in samples with a calcium peroxide content of 6% or higher. These results indicate that an optimized PLA filament with a 6% calcium peroxide content holds great promise for improving bone generation through bone cell oxygenation and resistance to bacterial infections.

## 1. Introduction

The shortage of organ donors is a significant challenge for global healthcare systems. The growing need for tissue and organ transplants has led to a significant gap between the availability of donors and the demand for transplants. This issue is particularly pronounced in the United States, where, as of March 2022, 106,097 people were waiting for organ transplants. Unfortunately, the shortage of donors results in a tragic loss of life. Current statistics indicate that, as of March 2022, an average of 17 deaths per day in the United States were attributed to delays in transplant surgery due to a lack of available organs [1].

Bone is an essential component of our skeletal system that provides structural support, protects vital organs, and enables movement. However, the human body’s ability to repair bone injuries is limited, making bone grafting a crucial procedure for bone fracture healing and regeneration. A bone graft is a surgical procedure in which bone tissue is transferred from one location in the body to another location where bone loss has occurred [2]. The use of autografts, which involves using the patient’s own tissue, is the current standard for bone fracture healing and regeneration. However, this method may not be practical in situations where the fractured bone is too large or precise shaping is needed, such as in facial bones [3]. Tissue engineering presents a potential solution to these challenges by creating a bone scaffold using stem cells, biocompatible materials, growth factors, and biodegradable materials to improve bone fracture healing [4]. These bone scaffolds are designed to supply the required physical support and foster tissue regrowth, leading to the recovery of functionality [5,6,7].

Recently, there has been a growing interest in fabricating bone scaffolds using 3D printing technologies. This approach allows the design of porous scaffolds with a specific exterior design and porous interior structure to achieve scaffolds with tailored functionality. This enables the scaffold to mimic the natural bone structure and support bone growth. Furthermore, 3D printing technology also allows for the large-scale production of scaffolds, making it more cost-effective and efficient [8]. Fused Deposition Modelling (FDM), a technique that utilizes filament polymers, is commonly adopted in tissue engineering due to its cost-effectiveness, accessibility, and availability [9]. Additionally, it offers a printing accuracy of +/−0.5 mm and can incorporate a wide range of biocompatible and biodegradable polymeric materials suitable for tissue engineering [9,10].

Materials such as polylactic acid (PLA, (C_3_H_4_O_2_)_n_), polycaprolactone (PCL, (C_6_H_10_O_2_)_n_), and poly(glycolic acid) (PGA, (C_2_H_2_O_2_)_n_) are commonly used in bone repairs, tendons, and skin, as they possess favorable characteristics [11,12]. PLA is particularly attractive due to its physical and mechanical properties, biocompatibility, and biodegradability, which can be affected by its molecular weight [13]. Its excellent properties make it useful in a wide range of biomedical applications [14]. Furthermore, PLA has been used in 3D printing and has been approved by the US Food and Drug Administration (FDA) for use in healthcare applications, such as biomedical scaffolding [15]. PLA composites have been explored by researchers to enhance the properties of pure PLA by incorporating micro- or nano-sized bioactive materials, such as hydroxyapatite (HA), to improve its mechanical strength and ability to integrate with surrounding bone tissue [16]. Chitosan is another material mixed with PLA to enhance its properties. PLA-chitosan-HA scaffolds with large pores were used to create a composite hydrogel that facilitated high levels of human stem cell osteogenesis. A composite of PLA, polycaprolactone, and titanium oxide has also been 3D printed to create a bone replacement with improved tensile strength and fracture strain [17].

Despite the promising results of tissue engineering research, it has had limited success in clinically treating small tissue defects of less than a few millimeters. The main reason for this is the lack of vascularization, which results in an insufficient oxygen supply [18]. Poor oxygen supply in engineered tissues is a major constraint in scaffold applications, as it is essential for the survival and growth of cells attached to the scaffold [19]. Researchers have developed scaffolds that generate oxygen from solid particles such as sodium percarbonate, magnesium peroxide, and calcium peroxide. These particles typically decompose and generate oxygen through hydrolysis, as shown in Equations (1) and (2) [20].

(1) $$\left(\mathrm{Calcium}\;\mathrm{peroxide}\right)\;C_{a}O_{2}\left(s\right)+{2H}_{2}O\;\to \;\mathrm{Ca}{\left(\mathrm{OH}\right)}_{2}\left(s\right)+H_{2}O_{2}$$

(2) $$2H_{2}O_{2}\;\to \;O_{2}+2H_{2}O\;\left(\mathrm{Catalase}\right)$$

Hydrogen peroxide, an intermediate product of the reaction, is thought to be a cytotoxic agent. Mammalian cells in the human body have mechanisms in place to decompose hydrogen peroxide into water and oxygen with the help of catalase enzyme and are generally able to tolerate low levels of hydrogen peroxide [21]. Due to their low toxicity, they can be used for tissue-engineering applications at concentrations that are well-controlled [22]. Zhang et al. [23] prepared polycaprolactone (PCL) mixed with CPO composite microspheres using three different methods. The resulting microspheres were found to support the ability of MIN6 cells, a pancreatic beta-cell line, to survive for a period of one week.

Recent literature suggests that 3D-printed oxygenation filaments in bone scaffolds can significantly improve bone tissue regeneration and healing. The filaments generate and release oxygen within the scaffold, providing an optimal environment for the scaffold-attached cells and promote their survival, proliferation, and differentiation. Additionally, the oxygen supply within the scaffold enhances the success of the scaffolding functionality by promoting vascularization, which is the formation of blood vessels within the scaffold and is crucial for the transport of oxygen, nutrients, and other substances to the cells. Three-dimensional printing technology is a powerful tool for the fabrication of these filaments and allows for precise control of the filament’s structure, composition, and size. This enables the creation of scaffolds that mimic the properties of natural bone, promoting better cell attachment and tissue regeneration. Overall, the use of 3D-printed oxygenation filaments in bone scaffolds shows great promise as a method for improving bone tissue regeneration and healing [19]. PLA/CPO is considered a promising composite material, yet research in this area is limited. A novel PLA/CPO composite filament was developed by utilizing wet solution mixing and hot melt extrusion in this research. The wet solution mixing method allows for the homogenous distribution of the CPO particles in the PLA matrix, while the hot melting extrusion process ensures that the filament has a consistent and uniform diameter. The prepared filaments were tested for 3D printing and analyzed using techniques including X-ray diffraction and scanning electron microscopy. Additionally, we also evaluated the oxygen release, porosity generation, and antibacterial activity of the filaments.

## 2. Results and Discussion

### 2.1. Presence of CPO and Printability of Filament

**Figure 1:**
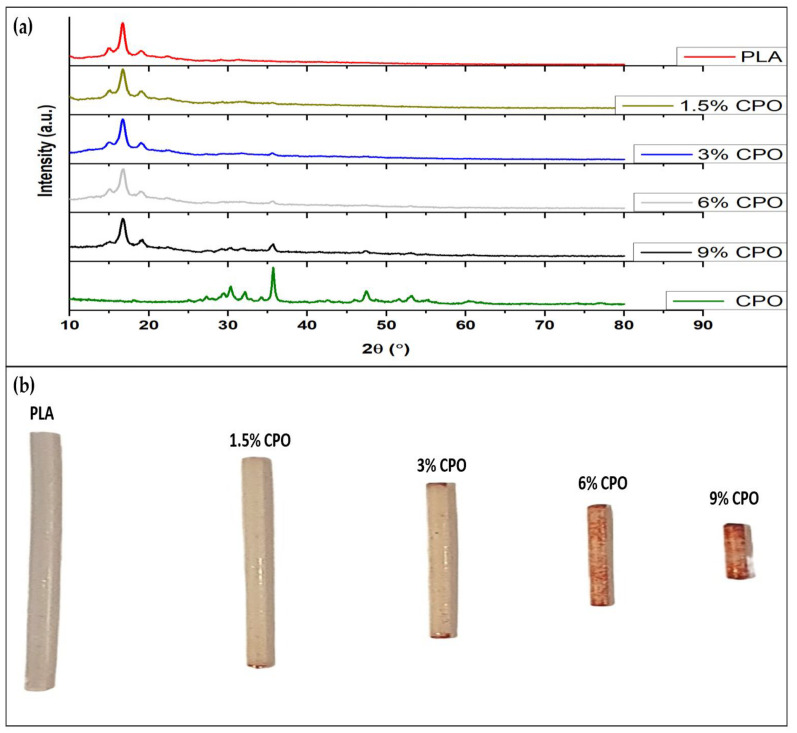
Calcium peroxide detection using (**a**) XRD and (**b**) Alizarin Red staining.

CPO particles within the fabricated filament were detected using XRD and Alizarin Red Staining solution. XRD was used to identify the crystalline phases in the composite filament and confirm the presence of CPO. Furthermore, the XRD was also used to investigate the effect of CPO solid loading on the crystal structure of the composite filament. The results demonstrate the presence of the CPO in each sample. The XRD pattern shown in Figure 1a for PLA highlights two characteristic peaks at 2θ 16.8° and 19.5°. For the pure CPO, several characteristic peaks appeared at 2θ 30.3°, 35.8°, 47.5°, 53.3°, and 60.7°. In all of the prepared samples, the PLA and CPO peaks were observed in the prepared composite. The intensity of the CPO peaks, which indicate the presence of CPO in the composite, varied depending on the concentration of the CPO in the composite filament. Samples with smaller CPO contents had smaller peaks than those with higher contents, indicating that increasing the CPO concentration in the composite filament also increases the presence of CPO.

Additionally, Alizarin red staining, which is a common method for detecting calcium ions, was applied to detect the presence of CPO in the samples by its ability to interact with calcium. This staining powder, Alizarin Red S, binds with calcium through its sulfonic acid and/or OH groups, making the calcium visible as a red stain. The composite filaments were stained red, and the intensity of the stain reflected the concentration of CPO embedded in the composite. The stain intensified from CPO-free to 9% CPO, further confirming the presence of CPO and the concentration in the composite filament, see Figure 1b. As CPO-PLA concentrations increased, the staining intensity also increased, indicating that CPO was successfully embedded into the PLA composite [24].

**Figure 2:**
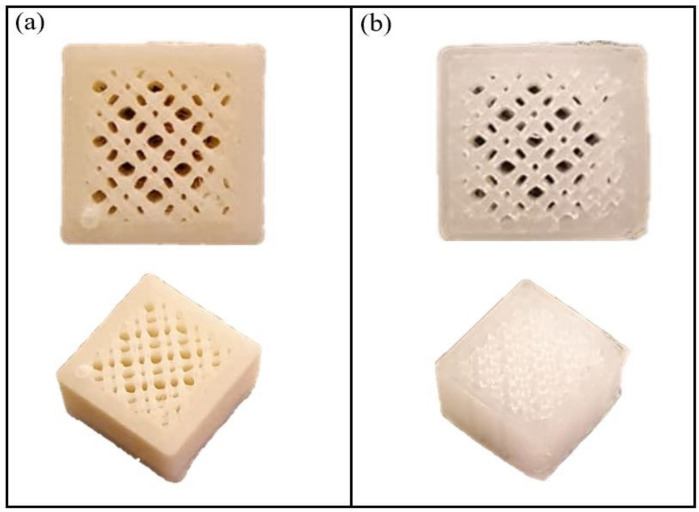
3D-printed scaffold by (**a**) 9% CPO composite filament (**b**) pure natural colour PLA.

The results indicate that the filaments were tested for printability, and a 3D cubed-shaped scaffold was successfully printed using the sample with the highest CPO ratio of 9%. The printing quality of this scaffold appears to be comparable to those printed with pure PLA, as shown in Figure 2. However, for scaffold applications, higher resolution is needed as significant differences in the mesh accuracy were observed when comparing the 3D model to the 3D-printed scaffold for both pure PLA and composite filaments. This suggests that further optimization of printing conditions is necessary to improve scaffold printing quality. Optimization can be achieved by varying the nozzle diameter, printing speed, and bed and nozzle temperature. Overall, these findings highlight the potential of using composite filaments for the 3D printing of scaffolds, but further improvements are necessary to achieve higher accuracy and resolution.

### 2.2. Filament Degradation, Oxygen and Calcium Ion Release

**Figure 3:**
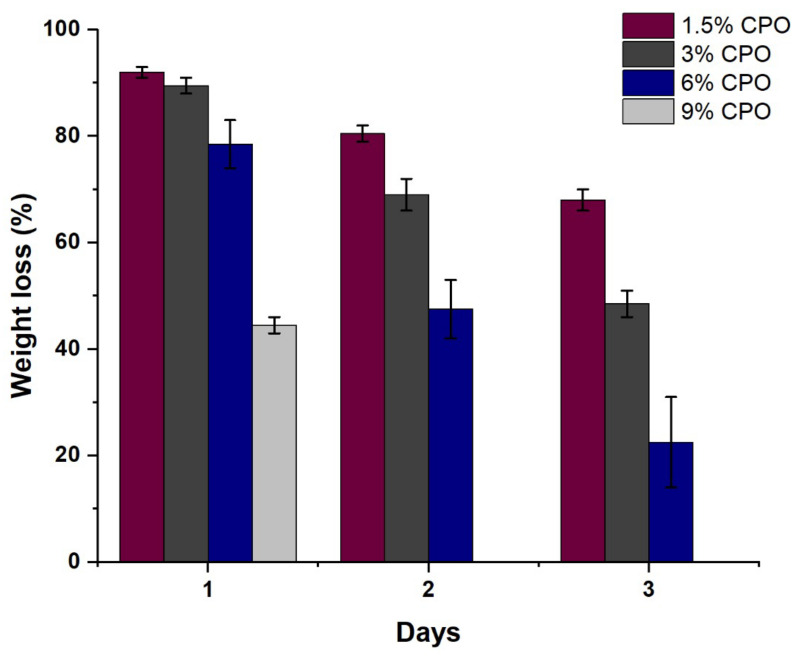
The weight loss percentage of samples over a three-day period (*n* = 3).

The aim of the degradation study was to investigate the degradation characteristics of a filament produced from a specific material. To expedite the degradation process, the samples were immersed in an alkaline solution and measured the percentage of weight loss over a specific period [25]. Previous studies have also used alkaline solutions to accelerate the degradation of polymer scaffolds. This approach was chosen to facilitate the degradation of both PLA and composite scaffolds as PBS, another degradation medium, can take more than six months to achieve significant weight loss [26]. By using an alkaline solution, we were able to compare the different CPO content in the filaments more effectively [26]. Figure 3 shows the degradation of the filaments with different CPO content over three days. It can be noted that the weight loss of 1.5% CPO samples was lower than 3%, 6%, and 9% CPO samples on days 1, 2, and 3. In general, there is a correlation between the degradation rate and CPO content in the composite filament. Increasing the CPO content increased the degradation percentage of the samples. It is worth noting that the 9% CPO sample completely degraded after 24 h. This was because the sample broke into large pieces and fell apart, unlike the other samples that retained their shape as a single solid piece while their weight decreased. The reason for the degrading behavior of the 9% CPO ratio can be understood when looking at the porosity section. Therefore, it appears that 9% CPO composites may not be an ideal ratio for the application of bone scaffolds.

**Figure 4:**
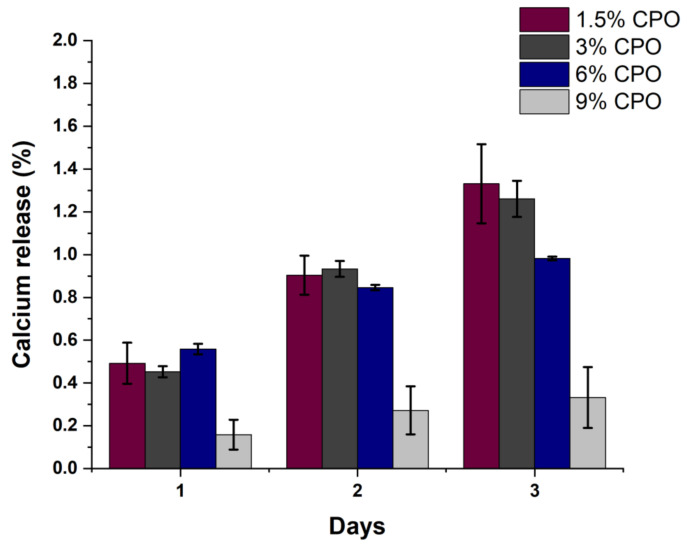
Calcium release percentage of samples over a three-day period (*n* = 3).

**Figure 5:**
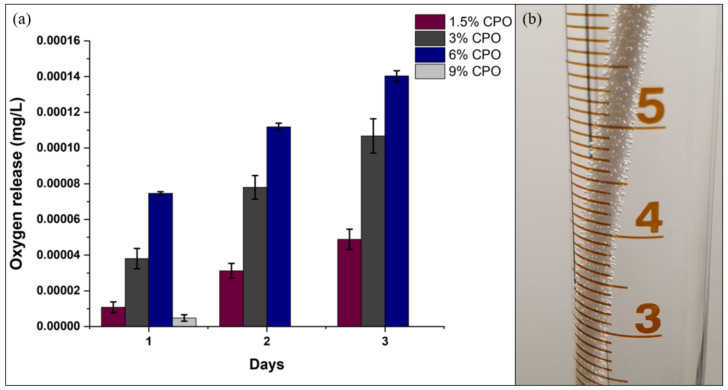
Oxygen release: (**a**) chart of oxygen release of samples over a three-day period (*n* = 3); (**b**) oxygen released during the degradation process evidenced in the release of gas bubbles from the filaments.

Figure 4 shows the cumulative percentages of calcium release for the samples from day one to day three. Percentages are calculated based on the total amount of CPO loaded for each sample. From the graph, we can note that the 1.5% sample released 1.3% of its total amount of CPO by day three, making it the fastest sample to release CPO compared to the other ratios. It is observable that there is an inverse relationship between the samples’ ratio and the release of CPO on the third day. The higher the CPO ratio, the slower the CPO content is released. Additionally, similar to the weight loss, the 9% CPO sample had a substantial decrease in the release of both CPO content and oxygen, as illustrated in Figure 5a.

The cumulative release of oxygen for all samples as a percentage of the loaded CPO is shown in Figure 5. Apart from the 9% CPO sample, all samples showed a linear positive correlation relation with respect to the CPO ratios. The oxygen release was greatest in 6% CPO at 0.00007mg/L from day one, followed by 3% CPO and 1.5% CPO. That can be due to the distribution and small sizes of CPO particles embedded in the PLA matrix, which accelerate the chemical reactions. On the other hand, although 9% CPO has the highest CPO content, we observed minimal oxygen release on day one (0.000005 mg/L) and no oxygen release on days two and three compared to the other CPO ratios. The acidity level of the samples remained stable throughout the three-day duration, with a slight decline of about 0.3.

Scaffolds for bone tissue that are made using materials that produce oxygen, such as PLA/CPO, can increase the oxygen concentration and result in enhanced cell survivability of the 3D-printed scaffolds. A biphasic calcium phosphate (BCP) scaffold, made up of 40% beta-tricalcium phosphate and 60% hydroxyapatite, was fabricated using the robocasting technique. Touri et al. fabricated biphasic calcium phosphate (BCP) scaffold composed of 60% hydroxyapatite and 40% beta-tricalcium phosphate using the robocasting technique. Although an alternative composite filament was investigated, the findings mirror that of our study, with the oxygen release behavior dependent on the concentration of CPO encapsulated in the PLA filaments. It was found that a 3% concentration of CPO was sufficient to produce desirable results in terms of promoting bone ingrowth, increasing the survival of osteoblast cells, and stimulating proliferation in low oxygen conditions [27].

Oversaturation remains a challenge in the preparation of composite filaments, with Zhang et al. observing that an environment with significantly oversaturated oxygen tension was created in the first 48 h in single-walled microspheres. In turn, this may result in the creation of a hyperoxia environment in cases where the oxygen demand by cells is not as high. Double-walled microspheres may better support the oxygen tension, but further research is warranted to determine their efficacy [28].

The available evidence suggests that oxygen-releasing materials are limited by their short release periods of 24 to 48 h [29,30,31]. However, the findings of our study indicate a more sustainable oxygen release over a three-day period, as seen in Figure 5. Similar oxygen release patterns were observed by Montazeri et al. and Lee et al. [32,33]. Future investigations should focus on the release periods of these materials and determine if they are of sufficient sustainability.

### 2.3. Antibacterial Activities

**Figure 6:**
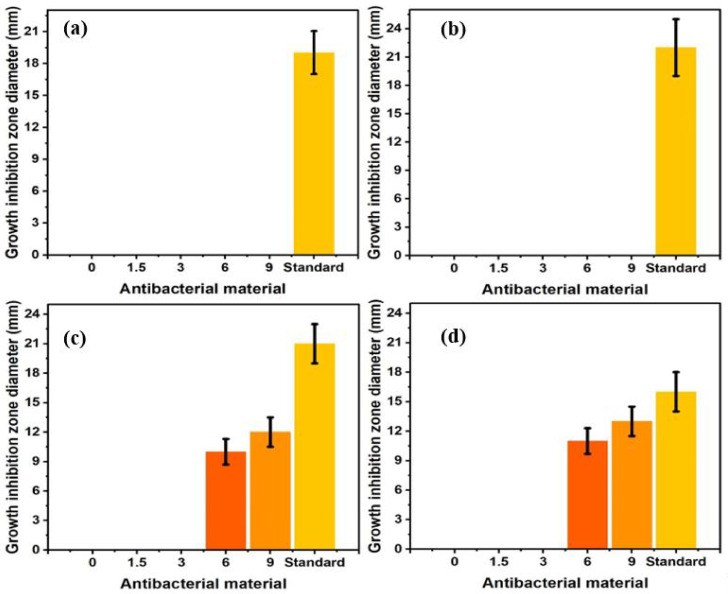
Antibacterial activity of composite scaffolds against (**a**) *Escherichia coli*, (**b**) *Pseudomonas aeru-ginosa*, (**c**) *Staphylococcus aureus*, and (**d**) *Methicillin-resistant Staphylococcus aureus* (*n* = 4). Note: The used standard is cefoxitin (FOX).

None of the samples displayed any inhibition zone when tested against *P. aeruginosa* bacteria and Gram-negative *E. coli* Figure 6a,b. However, when samples were tested against the Gram-positive bacterium *S. aureus* and MRSA, which has a higher resistant [32], some samples exhibited a clear inhibition zone [33,34].

As shown in the graphs in Figure 6c,d, the CPO-free, 1.5% CPO, and 3% CPO scaffolds did not produce any inhibition zone against *S. aureus* and MRSA, indicating that these PLA scaffolds with low CPO content had no effect on the bacteria. A significant inhibition zone was seen at 6% CPO and 9% CPO, indicating that increasing the CPO content to 6% or higher resulted in an antibacterial activity against Gram-positive bacteria, which is consistent with previous research [35], indicating that the antibacterial activity may be attributed to the residual hydrogen peroxide, which is a by-product of CPO decomposition [36].

### 2.4. Porosity

**Figure 7:**
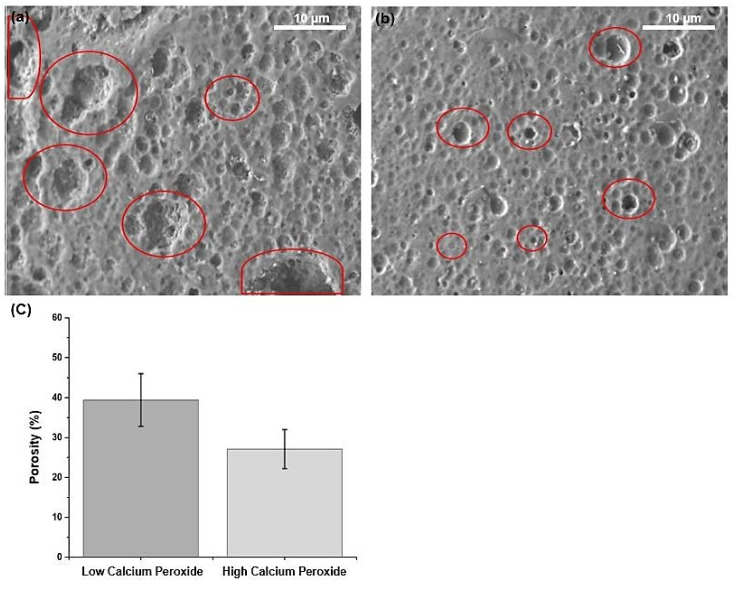
The effect of calcium peroxide on polylactic acid porosity. SEM images of the filament surface after degradation, showing porosities at (**a**) high and (**b**) low calcium peroxide at a magnification of ×2500 and an energy of 5.0 keV. (**c**) a semi-quantitative analysis of filament porosity based on image analysis (*n* = 3).

Porosity is among the most critical properties of biomaterials, particularly in tissue engineering applications, as it is correlated to the biomaterials’ swelling capability. Figure 7a,b shows the SEM observation of the samples after 20 min of degradation. The scanning was carried out to show the porosity size and distribution on the surface of the material, as after a few hours of degradation, the sample will have inner and merged porosities, see the red circles in Figure 7a,c. In all of the samples, there were many pores of different sizes all over the surface. This is because the dissolved calcium ions in the PLA/CPO composite left pores behind. In addition, the SEM image shows that several large particles of CPO did not sufficiently disperse in the composite with a high CPO concentration Figure 7a. Conversely, at CPO concentrations lower than 6%, small-sized pores were found (Figure 7b) with a porosity of approximately 40% of the selected area, as shown in Figure 7c. It is vital to note that if two pores overlap on the surface of a composite filament, ImageJ considers them a single pore. Thus, the variation in porosity content is related to CPO concentration and may allow for even finer-tuned and controlled porosities in the introduced biomaterial.

**Figure 8:**
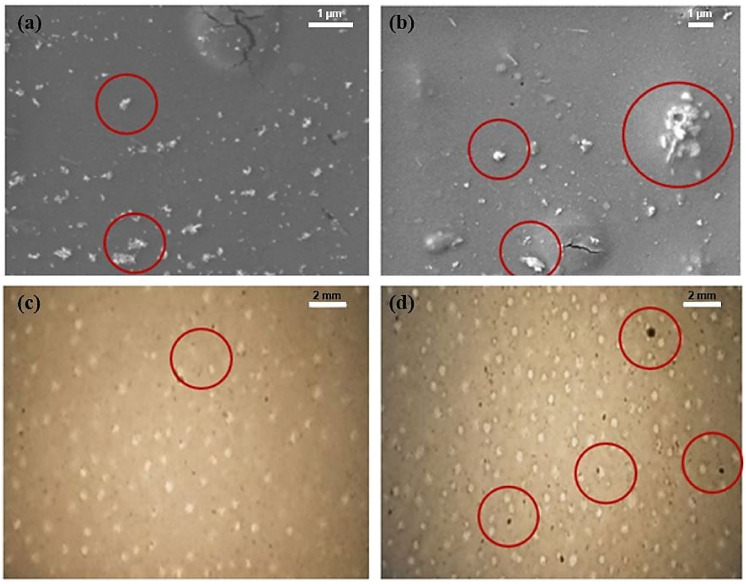
Undissolved calcium peroxide particles: (**a**) SEM image of low calcium peroxide concentration at a magnification of ×15,000 and an energy of 5.0 keV; (**b**) SEM image of high calcium peroxide concentration at a magnification of ×7500 and an energy of 5.0 keV. Optical image of the composite at (**c**) low and (**d**) high calcium peroxide concentration at a magnification of ×15.

In Figure 8a,b, the samples were investigated under the microscope using SEM with red circles representing the CPO particles. These images collectively show the CPO particles embedded within the PLA matrix. CPO particles are observed in the images of the samples as white spots. Unexpectedly, we found that 9% CPO released less oxygen and less calcium than the other CPO concentrations. We anticipated observing the opposite result as the outcomes of the antibacterial and red staining methodology contradicts these findings and confirm the presence of calcium within the sample. The SEM images in Figure 8a,b show a large number of CPO particles that are agglomerated together and not dispersed within the composite solution. These particles are relatively large in size, indicating that there is insufficient dispersion of the CPO within the composite solution. Moreover, Figure 8c,d highlights a larger number of particles of undispersed calcium peroxide (black dots) in the PLA polymer. Therefore, we estimated that at 9% CPO, the composite was already saturated, with saturation reached after 6% CPO.

## 3. Materials and Methods

### 3.1. Materials

For the study, 1.75 mm-diameter polylactic acid (PLA) filaments, molecular weight Mw 60,000, were obtained from (Shenzhen eSUN Industrial Co., Hubei, China). Calcium peroxide (CPO) with a particle size of −200 mesh and a purity level of 75% was procured from (Sigma-Aldrich, Saint Louis, MO, USA), a leading supplier of research chemicals and laboratory equipment. Additionally, pure natural bulk enzyme catalase powder (50,000 µ/g) was purchased from (Enzymes.bio, Wellington, New Zealand). Lastly, dichloromethane (DCM) was obtained from (Sigma-Aldrich, Saint Louis, MO, USA).

### 3.2. Preparation of PLA/CPO Filaments

**Figure 9:**
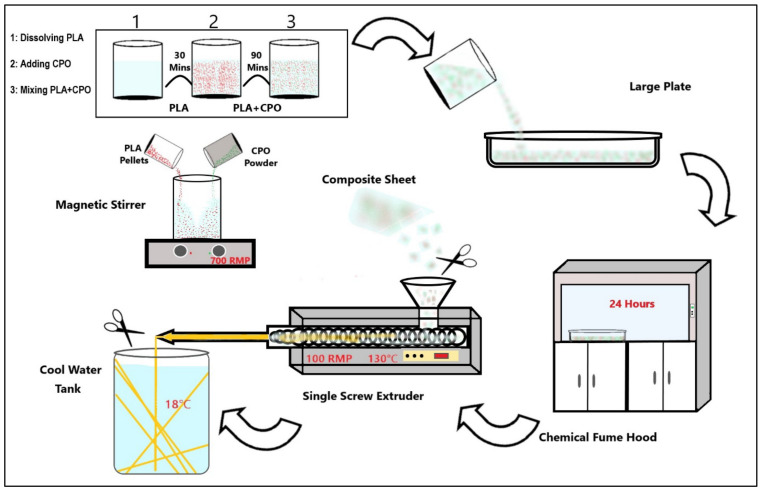
Schematic illustration of composite filament production.

The process of preparing the PLA/CPO composite filament starts by cutting PLA into 20 g pieces and dissolving it in 100 mL of DCM for 30 min at room temperature with a magnetic stirrer at 700 rpm, see Figure 9. Once the PLA samples were fully dissolved, various ratios of CPO were added to the mix while stirring vigorously for 90 min. The mixture was then poured onto a large plate and left to dry fo r 24 h. After drying, the composite was cut and loaded into a hot melting extruder. A composite material was extruded using a single screw extruder with a nozzle diameter of 2 mm from (King Abdelaziz University, King Abdelaziz, Saudi Arabia). The extrusion process was carried out at a nozzle temperature of 140 °C and an extruding speed of 2.5 cm/s. This resulted in a filament diameter of 1.75 mm, which is suitable for use in a commercial 3D printer. A diagram showing the process is depicted in Figure 1 and the prepared samples list is provided in Table 1. 

### 3.3. 3D Printing of Bone Scaffolds

To examine the generated filaments, a scaffold was printed using a commercial Fused Deposition Modelling (FDM) 3D printer—the Creality Ender 3 Pro model manufactured by (Shenzhen Creality 3D Technology Co., Ltd., Shenzhen, China). The printing process was carried out with the highest CPO ratio (9%) at a temperature of 200 °C and a speed of 75 mm/s. The scaffold was printed successfully in a box shape with dimensions of 8 × 8 × 8 mm for length, width, and height. The scaffold had a porosity of 25% with a pore size of 0.60 mm.

### 3.4. Characterisations

The composite filament’s surface and cross-sectional micrographs were captured using field emission scanning electron microscopy (FESEM) (FESEM, JEOL JSM 7600F, Tokyo, Japan). The imaging was performed at an acceleration voltage of 5 keV, and gold sputter coated prior to imaging to enhance the conductivity and provide better resolution. The images were used to study the morphology of the filament and to confirm the dispersion of the CPO particles in the PLA matrix. Additionally, optical images of composite filaments surface were captured using a Canon 1000D camera. This provided a visual representation of the filament’s surface and helped in identifying any morphological changes. Additionally, the XRD was carried out using Cu Ka radiations ULTIMA IV XRD systems (from Rigaku, Japan). The analysis of material elements was conducted using ICDD, DB card No. 01-071-4107. Additionally, Alizarin Red Staining solution was used to detect CPO particles within the fabricated filament.

To prepare the solution, 0.40 g of the Alizarin Red Staining powder was dissolved in 20 mL of distilled water with the aid of a stirrer. The samples were first soaked in an alkaline solution for 20 min and then left to soak in the same solution for 20 h in a dark environment.

### 3.5. Degradation and Oxygen Release

The degradation of the filament was examined by measuring the mass loss over time. A solution of 2 g of sodium hydroxide was made by dissolving it in 50 mL of distilled water to accelerate the degradation process. Each filament weighing 10 g was immersed in an alkaline solution at a room temperature of 18 °C, and the weight loss and calcium ion release were measured every 24 h during the degradation process for three days. The weight loss of the samples was calculated using Equation (3). The calcium ion release was observed using an ICP optical emission spectrometer (ULTIMA 2, HORIBA SCIENTIFIC Ltd., Kyoto, Japan).

(3) Weight=(Wt/Wo)×100%

where Wt represents the sample weight after degradation, and Wo represents the initial sample weight.

To measure the oxygen released by the filament, the samples were placed in a phosphate-buffered saline (PBS) solution with a pH of 7.4, and their oxygen levels were measured using an oxygen sensor. In addition, 10 mg of catalase were added in the solution.

The acidity level (pH) of the samples were measured every 24 h using a digital pH meter.

### 3.6. Porosity Measurements

The porosity was characterized using field emission scanning electron microscopy (FESEM) (FESEM, JEOL JSM 7600F, Tokyo, Japan) with an accelerating voltage of 5 keV. For each sample, three SEM images were captured from random spots. From the obtained SEM images, the porosity percentage of the total area was calculated using ImageJ (version 1.53f51). The SEM images were converted to 8-bit binary (white and black) images for ImageJ analysis, where the white spots indicate the solid surface and the black spots represent the pores.

### 3.7. Antibacterial Activity

The antimicrobial performance of the composite filaments was examined using disk diffusion tests. We utilized the following bacteria strains in the study: *Escherichia coli* ATCC 11,775 (*E. coli*), *Staphylococcus aureus* ATCC 12,600 (SA), Pseudomonas aeruginosa ATCC 9027 (PA), and Methicillin-resistant *Staphylococcus aureus* ATCC 33,591 (MRSA). We cultivated these bacteria strains on blood agar and stored them on Mueller Hinton agar (MHA) (Condalab, Madrid, Spain) plates in the refrigerator at 4 °C for later use. When the experiments were performed, the bacteria strains were activated in Mueller Hinton broth (MHB) (Scharlau, Barcelona, Spain); after measuring their turbidity (0.1 ± 0.02), they were spread on the MHA plates via swab cotton. The composite filaments were prepared in a disk shape and then fixed on the surface of the MHA plates containing the bacteria strain beside a standard 30 μg cefoxitin antibiotic disc (FOX) from Mast (Mast Group Ltd., Bootle, UK). Then, all MHA plates were incubated at 37 °C for 24 h. Finally, we measured the diameters of the bacteria growth inhibition zone; the antibacterial performances of the prepared composites were compared with the standard antibiotic FOX disks.

### 3.8. Statistics Analysis

Each test was conducted in triplicate, and a mean value was calculated. The data are calculated as the mean with a ± standard deviation (SD). OriginPro 8.0 software (OriginLab, Northampton, MA, USA) was employed to analyze the data.

## 4. Conclusions

The field of bone tissue engineering is expanding and focuses on developing new techniques and materials to repair or replace damaged or diseased bones. One promising area of research in this field is the use of 3D printing techniques to create composite filaments for use in bone tissue engineering. PLA is a biodegradable and popular polymer for tissue engineering applications due to its preferable biocompatibility and mechanical properties. CPO, on the other hand, is a material that can release oxygen and calcium ions when it comes into contact with water. This property makes it a useful component in bone tissue engineering, as the increased oxygen levels and calcium ions can help to promote bone growth and healing. This study introduces a novel PLA/CPO composite filament created through wet solution mixing and hot melting extrusion, a process that can control the composition and morphology of the filament. The study examined the effect of varying CPO content on filament performance. The results showed that a 6% CPO ratio provides optimal calcium and oxygen release. However, when increasing CPO content, the composite filament displays poor dispersion of large, agglomerated particles, which can affect its mechanical properties. The composite filament with CPO content above 6% showed excellent antibacterial activity, which can be useful in preventing infections in bone tissue engineering applications. Furthermore, an in-depth study about cytocompatibility is currently being performed and will be published in a separate paper.

## Figures and Tables

**Table 1:** PLA/CPO ratios.

Sample No.	Samples Name	PLA (%wt./v)	CPO (%wt./v)
1	0% CPO	100	0
2	1.5% CPO	98.5	1.5
3	3% CPO	97	3
4	6% CPO	94	6
5	9% CPO	91	9

## Data Availability

Data is contained within the article.
